# Validation of a new commercial serine protease artificial digestion assay for the detection of *Trichinella* larvae in pork

**DOI:** 10.1016/j.fawpar.2018.04.001

**Published:** 2018-04-09

**Authors:** Alvin Gajadhar, Kelly Konecsni, Brad Scandrett, Patrik Buholzer

**Affiliations:** a Parasitix Lab Services, Innovation Place, Saskatoon, Sask. S7N 3R2, Canada; b Centre for Foodborne and Animal Parasitology, Canadian Food Inspection Agency, 116 Veterinary Road, Saskatoon, Sask. S7N 2R3, Canada; c Thermo Fisher Scientific, Wagistrasse 27A, 8952 Schlieren, Switzerland

**Keywords:** Pork, Food safety, PrioCHECK Trichinella AAD assay, Pepsin/HCl digestion method, Diagnostic assay, Validation

## Abstract

*Trichinella* is a zoonotic nematode parasite transmitted by the ingestion of raw or under-cooked meat. Control of the parasite is essential to facilitate public health and trade in products from susceptible food animals, including pork and horse meat. The standard method for detecting *Trichinella* muscle larvae uses pepsin enzyme and hydrochloric acid (HCl) in an artificial digestion procedure. A new artificial digestion assay using serine protease was recently developed and commercialized (PrioCHECK™ Trichinella AAD) for the detection of *Trichinella* larvae in the muscle of infected animals. The assay uses no hazardous substances such as HCl or pepsin. Activation of the enzyme requires an elevated digestion temperature of 60 °C which kills the parasite and reduces the risk of contaminating the environment with *Trichinella*. Compared to the pepsin/HCl method, digestion time for the PrioCHECK Trichinella AAD assay is reduced by a third. A recent study demonstrated these features of the new assay and its suitability for digesting various muscles from domestic and wild animals. To further validate the assay's performance relative to the conventional pepsin/HCl digestion method several comparative studies were conducted using samples from different muscle sites spiked with low levels of encapsulated first stage *Trichinella* larvae (L1). Multiple muscle samples were collected from diaphragm, tongue, masseter, and loin of 3–4 month old pigs. Samples were spiked with 3, 4, 5, or 25 *Trichinella spiralis* L1. A total of 320 meat samples of 100 g each were used to compare the diagnostic proficiency of the PrioCHECK Trichinella AAD assay with the pepsin/HCl digestion method. Comparative and validation data produced from these studies showed that both methods are capable of consistently detecting *Trichinella* in 100 g samples which contained as few as 3 L1 or 0.03 larvae per gram of meat. Overall, the PrioCHECK Trichinella AAD assay performed satisfactorily according to international guidelines of the World Organization for Animal Health (OIE), European Union (EU) and International Commission on Trichinellosis (ICT) for the detection of *Trichinella* infection in pork.

## Highlights

• A new serine artificial digestion assay has been validated to test pork for *Trichinella*.
• The assay detected *Trichinella* from all 160, 100 g samples containing 3, 4, 5, or 25 larvae
• As few as 0.03 larvae per gram were detected using spiked samples from four muscle sites
• PrioCHECK Trichinella AAD and pepsin/HCl methods are comparable for detecting *Trichinella* in pork

## Graphical abstract

**Figure:**
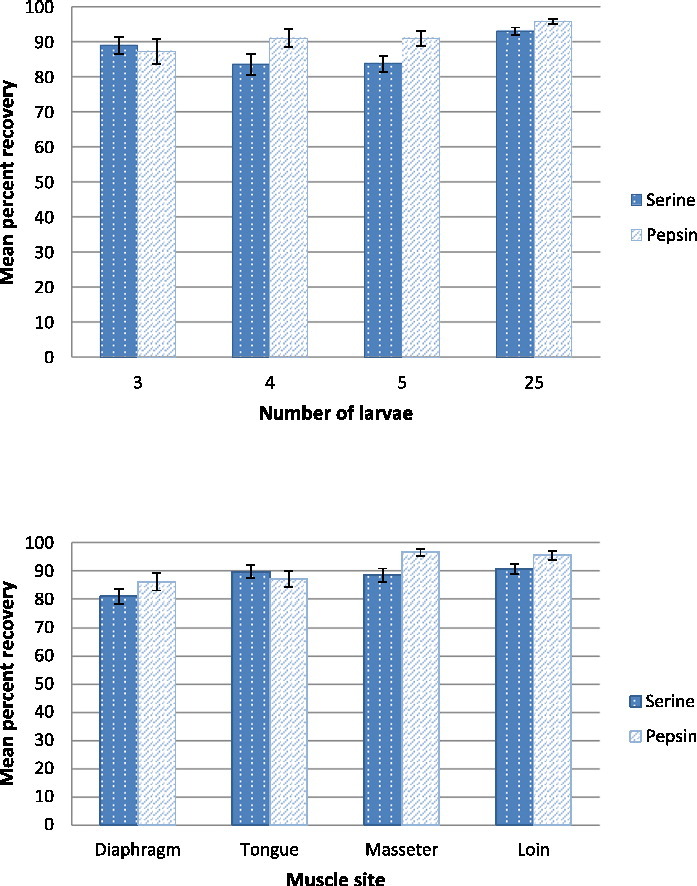

## 1 Introduction

*Trichinella* is a meat-borne nematode parasite that infects many species of animals. It is prevalent in wild carnivores and omnivores, with potential for spillover between susceptible species of wildlife and livestock (Pozio, 2015). Historically there has been a close association between *Trichinella spiralis* and pigs, but other species of the parasite can also infect porcine hosts (Pozio and Murrell, 2006). Raw or under-cooked pork has been a common source of transmission to humans. Although *Trichinella* infection is not recognized as a cause of disease in animals, any species of the parasite can cause mild to serious illness in humans, and in some cases even death (Gottstein et al., 2009). In a recent global ranking of foodborne parasites, *T. spiralis* was listed as the most important parasite for trade, and 9th in terms of public health (FAO/WHO, 2014). Adequate cooking of pork is usually recommended for protecting consumers from *Trichinella* infection. Other control measures are not as straight forward and may include freezing or curing to kill the parasite or biosecurity and management production practices to prevent exposure of pigs (Gamble et al., 2000; Alban and Petersen, 2016; ICT, 2016). A common tool for establishing or monitoring the effectiveness of control measures is the artificial digestion method which is used to detect first stage *Trichinella* larvae in muscle tissues of host species. In the EU alone, meat samples from millions of pigs are tested annually for the presence of *Trichinella* (Pozio, 2014).

The traditional artificial digestion assay uses pepsin enzyme activated by hydrochloric acid (HCl) and incubation, followed by sedimentation and microscopy for the detection of *Trichinella* larvae in meat, and has been described as a standard method (ISO 18743:2015). Although the assay is widely employed as a diagnostic tool for detecting *Trichinella* infection, it is more commonly used for regulatory certification related to trade and food safety to ensure negligible risk of *Trichinella* infection. The artificial digestion method processes individual samples or pools of samples weighing up to 100 g (ICT, 2014; OIE, 2017). A serine protease artificial digestion assay (PrioCHECK Trichinella AAD) was recently developed and evaluated using limited numbers of muscle samples from domestic and wild animals for the detection of *Trichinella* (Konecsni et al., 2017). The results for pork indicated test performance that was comparable with the standard pepsin/HCl digestion method, and confirmed lab advantages of convenience, safety and speed.

Reliable performance that is fit for purpose requires method validation, quality assurance measures, and suitable samples. The present large scale study was designed to generate experimental data to validate the performance of the PrioCHECK Trichinella AAD assay for detecting *Trichinella* in fresh (unfrozen) pork tongue, diaphragm, masseter and loin to meet various international standards for trade and food safety.

## 2 Materials and methods

### 2.1 Experimental design

To demonstrate the performance of the PrioCHECK Trichinella AAD assay a total of 160 pork samples from four muscle sites were spiked with proficiency samples containing encapsulated *T. spiralis* first stage larvae (L1) and tested for parasite recovery. An additional 160 matching samples were tested using the traditional pepsin/HCl digestion method for detecting *Trichinella* in pork.

Specifically, ten fresh muscle samples collected from each of diaphragm, tongue, masseter, and loin spiked with 3, 4, 5, or 25 *T. spiralis* L1 were tested using the PrioCHECK Trichinella AAD assay (Table 1). A matching set of spiked samples was also tested by the traditional pepsin/HCl digestion method. Prior to spiking, a 100 g sample from each muscle site used was tested by the pepsin/HCl method to ensure *Trichinella*-negative status of the source tissue.

**Table 1:** Numbers of 100 g pork samples collected from four muscle sites spiked with encapsulated larvae (L1) of *Trichinella spiralis* and tested by the serine digestion assay (PrioCHECK Trichinella AAD) or standard pepsin/HCl digestion method.

Muscle	PrioCHECK Trichinella AAD				Pepsin/HCl			
	3 L1	4 L1	5 L1	25 L1	3 L1	4 L1	5 L1	25 L1
Diaphragm	10	10	10	10	10	10	10	10
Tongue	10	10	10	10	10	10	10	10
Masseter	10	10	10	10	10	10	10	10
Loin	10	10	10	10	10	10	10	10
Total	40	40	40	40	40	40	40	40

### 2.2 Sample collection and preparation

All pork muscle samples used in this study were collected from a local commercial abattoir. Sufficient samples of each of the four muscle sites were collected from as few carcasses as possible to minimize variability. *Trichinella*-free muscles were trimmed to remove visible fat and fascia, vacuum packed and kept at 4 °C for use within 6 weeks. After confirming the *Trichinella*-negative status of each site, 100 g muscle samples containing proficiency samples with precise numbers of embedded encapsulated L1 were prepared as previously described (Forbes et al., 1998; Konecsni et al., 2017). The experimental infection and use of rats to produce *T. spiralis* L1 for the proficiency samples were approved by the University of Saskatchewan Animal Care Committee in accordance with the guidelines of the Canadian Council on Animal Care.

### 2.3 Digestion assays

All aspects of the digestion assays used in this study, including sample preparation and stereo-microscopic examination, were performed by trained and certified analysts in a laboratory accredited or certified for *Trichinella* digestion testing according to guidelines of ICT and ISO 17025 (Gajadhar and Forbes, 2002; ICT, 2014). The analysts had many years of experience conducting *Trichinella* testing for routine diagnostics and research on pork, horse meat and meat of wild animals.

The commercial serine digestion assay (PrioCHECK Trichinella AAD, Thermo Fisher Scientific) for the detection of *Trichinella* L1 in muscle samples employs a serine protease enzyme at 60 °C (±2 °C) for 20 min to digest muscle tissue and kill released L1 that are isolated and concentrated in subsequent sedimentation processes and detected by stereo-microscopic examination. The assay was used according to the manufacturer's instructions, with the following exceptions, based primarily on the previous study by Konecsni et al., 2017. In the present study, 1000 mL of water was used to rinse the remnants in the digestion beaker (300 mL rinse) and the sieve (700 mL rinse) into the separatory funnel. This resulted in a final volume of 3 L for a primary 30 min sedimentation in the separatory funnel. A 4 L separatory funnel was used to accommodate this 3 L volume. Further details of the assay were followed as recently published (Konecsni et al., 2017).

The method used for comparative purposes was the traditional magnetic stirrer pepsin/HCl artificial digestion method that is widely used in many countries for the detection of L1. A solution consisting of 1% liquid pepsin (2800–3500 FC units/mg, American Laboratories Inc., Omaha, NE, USA) and 1% HCl at 44 °C (±3 °C) for 30 min was used for digestion. Isolation and concentration of L1 were conducted by sequential sedimentation in an apparatus with double separatory funnels as previously described (Forbes and Gajadhar, 1999; OIE, 2017). The main differences between the pepsin/HCl digestion method and the PrioCHECK Trichinella AAD assay are listed in Table 2.

**Table 2:** Basic differences and similarities between the standard pepsin/HCl digestion method and the serine assay PrioCHECK Trichinella AAD, as used in this study for the detection of *Trichinella* larvae in meat samples.

Method	Enzyme	Digestion temperature	Digestion time	Digestion volume	Rinse volume added to settling funnel	Larvae recovered
PrioCHECK Trichinella AAD	Serine protease	60 °C (±2 °C)	20 min	2 L	1 L	Dead
Standard pepsin/HCl digestion	Pepsin protease	44 °C (±3 °C)	30 min	3 L	1 L	Live/dead

The final sediment collected from each of the two methods was transferred into a gridded Petri dish and left undisturbed for at least 1 min to allow settling of larvae to the bottom. A stereo-microscope at 10× or 16× magnification was used to systematically examine the entire gridded dish for the presence of *Trichinella* larvae. If necessary, a higher magnification was used to differentiate L1 from artifacts (Gajadhar et al., 2009; ICT, 2014). During the study, 10 spiked samples had to be replaced from the original source and retested due to inadvertent freezing during storage or spillage from a malfunctioning stop cock on a separatory funnel.

### 2.4 Statistical analysis

Mean percent larval recoveries for each spike level and muscle site were obtained by dividing the total number of larvae detected for each set of samples by the number of samples. Standard error of the mean was calculated for each sample set. Statistical significance was determined using one way ANOVA and the Tukey's multiple comparison post-hoc test; a *P*-value < 0.05 was deemed significant. All statistical analyses were performed using Prism version 4 software.

## 3 Results

### 3.1 Overall results

Both *Trichinella* testing procedures used in this study performed satisfactorily and without any problems. Occasionally, an additional clarification step was required in both methods to remove debris from the sediment that might obscure the detection of larvae during microscopic examination; this requirement was mostly for the PrioCHECK Trichinella AAD assay (data not shown). Motile as well as non-motile larvae were recovered by the pepsin/HCl method whereas only non-motile L1 were recovered by the PrioCHECK Trichinella AAD assay (data not shown).

**Fig. 1:**
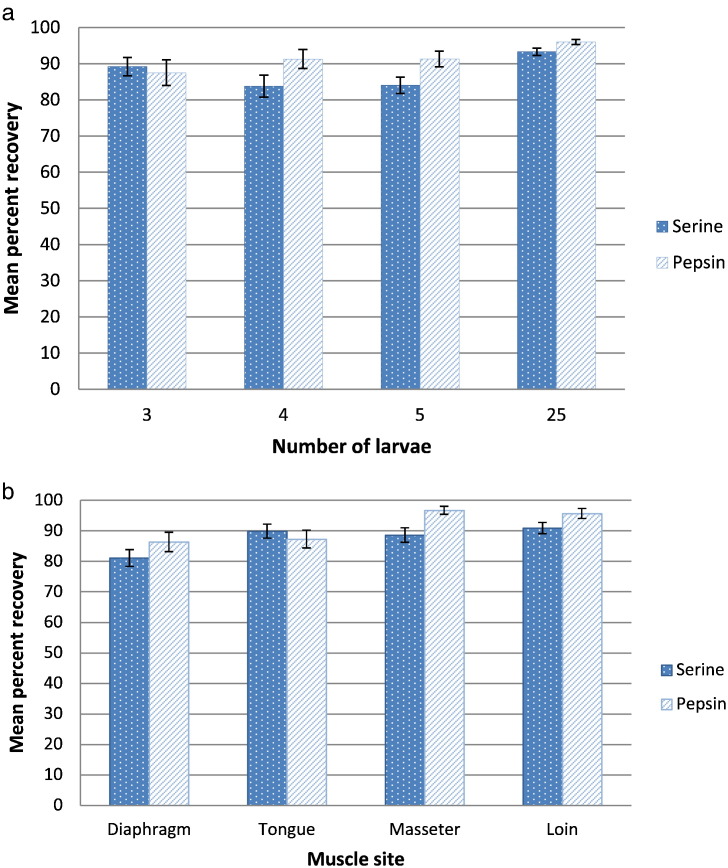
a. Mean percent larval recovery and standard error, by spike level for digestion by serine compared to pepsin/HCl using combined results obtained from a total of 160 pork samples per method. The samples consisted of diaphragm, tongue, masseter and loin that were each spiked with 3, 4, 5, or 25 encapsulated larvae of *Trichinella spiralis*. b. Mean percent larval recovery and standard error, by muscle site for digestion by serine compared to pepsin/HCl using combined results from 160, 100 g pork samples per method. Samples of diaphragm, tongue, masseter and loin were each spiked with 3, 4, 5, or 25 encapsulated larvae of *T. spiralis*.

**Fig. 2:**
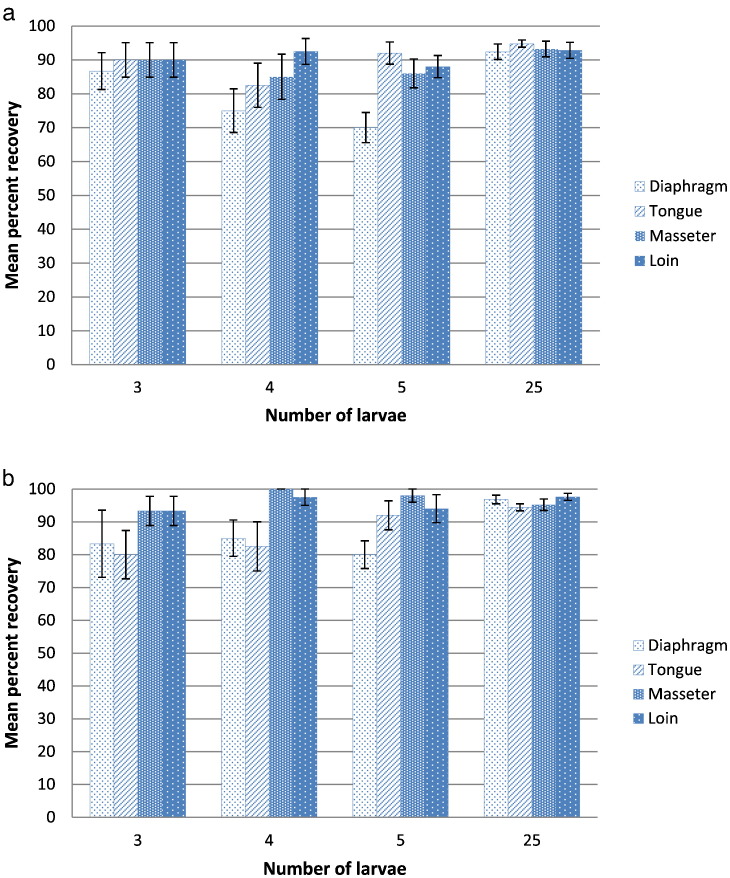
a. Results of the serine digestion PrioCHECK Trichinella AAD assay showing mean percentage and standard error of larval recovery by spike level from samples of pork diaphragm, tongue, masseter and loin that were spiked with 3, 4, 5 or 25 *T. spiralis* larvae. b. Results of pepsin/HCl digestion method showing mean percentage and standard error of larval recovery by spike level from samples of pork diaphragm, tongue, masseter and loin that were spiked with 3 4, 5 or 25 *T. spiralis* larvae.

**Fig. 3:**
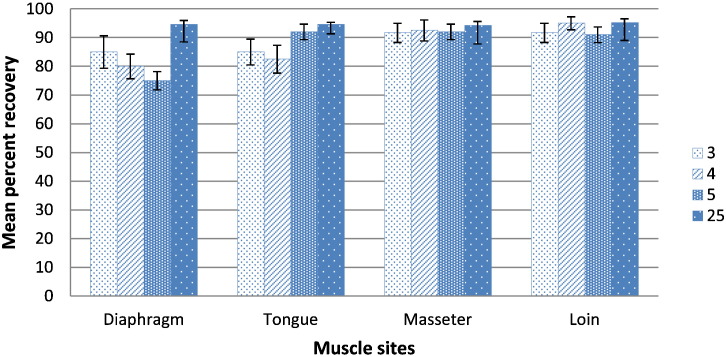
Mean percentage and standard error of larval recovery by muscle site (diaphragm, tongue, masseter and loin) and spike levels (3, 4, 5 or 25 L1), using combined results of both digestion methods (pepsin/HCl and PrioCHECK Trichinella AAD).

**Fig. 4:**
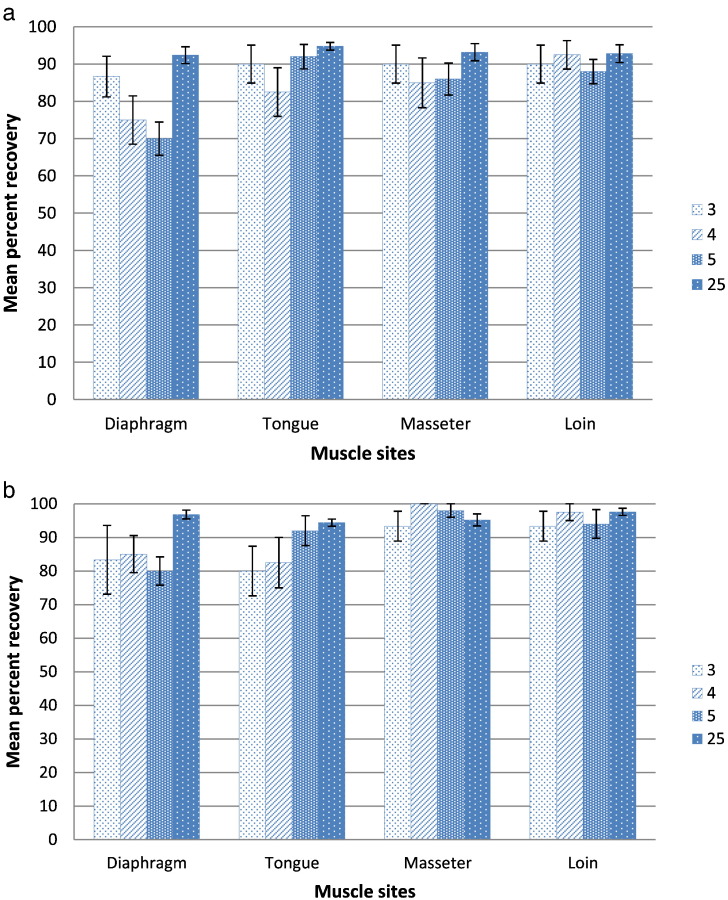
a. Results of the serine assay PrioCHECK Trichinella AAD showing mean percentage and standard error of larval recovery by muscle site (diaphragm, tongue, masseter and loin) and number of spiked larvae (3, 4, 5, or 25 L1). b. Results of pepsin/HCl method showing mean percentage and standard error of larval recovery by muscle site (diaphragm, tongue, masseter and loin) and number of spiked larvae (3, 4, 5, or 25 L1).

Both methods detected *Trichinella* larvae in all spiked samples, regardless of the number of L1 used for spiking or the muscle site (Fig. 1, Fig. 2, Fig. 3, Fig. 4). None of the samples tested by either assay yielded larval recoveries >100% of the spiked numbers. The numbers of larvae recovered by the pepsin/HCl method were usually slightly higher, although there was no statistically significant difference between assays within spike level or muscle site (Fig. 1, Fig. 2, Fig. 4). For both methods, the minimum mean recovery rates by spike number or muscle site were always >80%, except for the 75.0% and 70.0% recoveries by the PrioCHECK Trichinella AAD assay from diaphragm samples spiked with 4 and 5 L1, respectively. When results were combined by muscle sites or spike level, samples spiked with 25 L1, and samples of masseter yielded the highest mean recoveries of 96.0% and 96.6%, respectively, by the pepsin/HCl digestion method (Fig 1a, b). The PrioCHECK Trichinella AAD assay had a marginally higher mean percent recovery from samples containing 3 L1 and samples of tongue, whereas the mean recoveries by the pepsin/HCl digestion method were higher for all other sample sites and spike levels (Fig. 1a, b).

### 3.2 Recovery of larvae according to spike level and tissue type in samples

The highest and most consistent mean rates of recovery (>90%) were obtained by both the PrioCHECK Trichinella AAD assay and the pepsin/HCl digestion method from all four muscle sites that were spiked at the 25 L1 level (Fig. 2a, b). Samples from the four muscles spiked with 3 L1 consistently yielded ≥86% recovery by the PrioCHECK Trichinella AAD assay, while the recovery by the pepsin/HCl method was lower (>80%) and more variable for tongue and diaphragm samples that were spiked at the same level. The percent recoveries by both methods for samples containing 4 or 5 L1 were generally reduced and more variable (Fig. 2a, b). Within each spike level, the diaphragm yielded the lowest mean percentage of L1 for the PrioCHECK Trichinella AAD, while with the pepsin/HCl method it was either tongue or diaphragm. However, the only mean values below 80% were the 70% and 75% recoveries obtained using the PrioCHECK Trichinella AAD for diaphragm samples containing 4 or 5 L1, respectively (Figs. 2a, 4a). For each method, these observed differences amongst muscle sites within spike level or spike levels within each muscle site were not statistically significant. The mean percent recovery of larvae by the PrioCHECK Trichinella AAD assay and the pepsin/HCl method according to muscle sites, and for the four spike levels, are shown in Fig. 4a and b, respectively.

### 3.3 Recovery of larvae according to combined results of both assays

The combined results of both methods yielded mean percent of larval recoveries that were generally lower and more variable for diaphragm and tongue samples with all levels of spikes except 25 L1 (Fig. 3), although this variability within muscle sites was not statistically significant. Except for diaphragm samples spiked with 5 L1, all muscle sites containing 5 or 25 L1 consistently yielded the highest mean percentages of larval recovery (Fig. 3).

## 4 Discussion

This report describes an experimental study for the validation of an alternative artificial digestion method to detect *Trichinella* in pork muscle samples. As far as we know, it is the largest such study to date using proficiency samples that are recommended for QA purposes (ICT, 2014). A total of 320 digestion assays were performed on 100 g pork samples derived from four muscle sites, including tongue, diaphragm, and masseter which are well recognized as preferential sites for *Trichinella* infection and for diagnostic sampling. The loin was also used because of ease of acquisition from carcasses and retail markets, and because it is widely used by consumers.

The present study used artificially prepared proficiency samples to ensure precise numbers of larvae in the samples tested, unlike many other validation studies that have used naturally infected meat containing uncontrolled numbers of larvae (Gamble, 1996; Forbes and Gajadhar, 1999; Leclair et al., 2003). Spike levels included 3, 4 and 5 L1 in accordance with the ICT recommendation of 3–5 L1 per proficiency sample for validating digestion methods (ICT, 2014). Also, identification of positive samples harbouring ≥1 larva per gram is generally accepted to demonstrate sufficient diagnostic sensitivity for the lowest larval burdens that may cause clinical disease in humans (Dupouy-Camet and Bruschi, 2007). The individual 100 g samples spiked with 3 L1 in this study represent a detection level (analytical sensitivity) of 0.03 larvae per gram of meat. Detection of 3 or 5 larvae in 100 g samples represents a much higher level of sensitivity than the 1 larva per gram recommended by regulatory and public health authorities for trade and food safety, respectively (ICT, 2016; OIE, 2017). However, it should be noted that the sensitivity of detecting an infected pig (diagnostic sensitivity) depends on the size of the corresponding sample tested, and is therefore lower for digestion assays of 100 g of pooled samples from multiple carcasses.

To further demonstrate the performance of the PrioCHECK Trichinella AAD assay and to validate its diagnostic capability for detecting *Trichinella* in pork, replicate samples were tested by the standard pepsin/HCl digestion method for comparison. Overall, the results of both methods indicated reliable detection of the parasite in every type of muscle tested and at all spike levels. This confirms previous data that the PrioCHECK Trichinella AAD assay adequately digests and isolates larvae from a variety of muscles, including tongue which has a high proportion of connective tissue. Within spike levels, the mean larval recovery between the two methods were not significantly different; the PrioCHECK AAD Trichinella assay had a slightly higher (2%) mean recovery rate for samples with a larval load of 0.03 LPG (spiked with 3 L1), whereas mean recovery rates for pepsin/HCl were slightly higher (3–5%) for samples containing 0.04–0.25 LPG (spiked with 4, 5, or 25 L1).

When mean larval recoveries between muscle sites were compared for each method, there was no significant difference. However, as shown by the combined results of both digestion methods (Fig. 3), the highest rates of larval recovery were consistently obtained from loin, as well as masseter samples. This was probably due to the lesser amounts of fat and fascia in this muscle site that facilitated more complete digestion, and a commensurate reduction in obstructive non-digestible tissues on the sieve. However, it should be noted that, regardless of muscle site, both digestion methods usually resulted in negligible amounts of non-digestible materials (data not shown) that were well under the 5% acceptable EU-recommended limit (European Commission, 2015). Nevertheless, as noted in this study and previously, additional sediment clarification steps may be required more often for the PrioCHECK Trichinella AAD assay than the pepsin/HCl method (Konecsni et al., 2017).

Based on previous studies of naturally infected animals, tongue and diaphragm have generally yielded the highest larval recoveries and are thus recommended as preferred sampling sites for *Trichinella* testing (Gamble et al., 2000; Kapel et al., 2005). However, it is not surprising that these predilection muscles did not consistently yield higher larval recoveries by either method in this study, as all samples were spiked and not naturally infected. The results from the present study which used spiked samples demonstrate that both digestion methods perform comparably on samples from all muscles that are adequately trimmed and processed according to validated procedures.

The extensive data presented in this study validate the performance of the PrioCHECK Trichinella AAD assay for testing fresh pork samples for *Trichinella* according to international guidelines, including those of the EU, ICT and OIE (European Commission, 2015; ICT, 2014; OIE, 2017). Although the rates of larval recovery were comparable to those obtained by the pepsin/HCl method, the PrioCHECK Trichinella AAD assay possesses technical and safety advantages, as well as some potential disadvantages relative to the standard pepsin/HCl method. The digestive enzyme serine is non-hazardous and safe for use by analysts, a third less time is required for the digestion process, and the risk of environmental contamination is mitigated by the elevated digestion temperature which is sufficient to kill any *Trichinella* larvae present (Konecsni et al., 2017). Some potential disadvantages which can be mitigated by adequate training include the elevated digestion temperature (+15 °C compared to pepsin/HCl method) requiring skin protection, decreased visibility of non-motile recovered larvae, and the occasional need for an additional clarification step. Nevertheless, these issues may be outweighed by specific conveniences and advantages of the kit over the pepsin/HCl method. The serine assay PrioCHECK Trichinella AAD is currently employed for both large-scale and low volume testing in several regulatory jurisdictions for various purposes including trade, food safety, and production of pigs in *Trichinella* negligible risk compartments.

## Conflict of interest

Patrik Buholzer is an employee of Thermo Fisher Scientific. All other authors declare no conflict of interest in this study.
